# Are interventions delivered by healthcare professionals effective for weight management? A systematic review of systematic reviews

**DOI:** 10.1017/S1368980021004481

**Published:** 2022-04

**Authors:** Tracy Epton, Christopher Keyworth, Joanna Goldthorpe, Rachel Calam, Christopher J Armitage

**Affiliations:** 1 Manchester Centre for Health Psychology, Division of Psychology and Mental Health, University of Manchester, Oxford Road, Manchester, M13 9PT, UK; 2 School of Psychology, University of Leeds, Leeds, UK; 3 Faculty of Health & Medicine, Lancaster University, Lancaster, UK; 4 Manchester University, NHS Foundation Trust, Manchester Academic Health Science Centre, Manchester, UK

**Keywords:** Weight management interventions, Weight loss interventions, Healthcare professionals, Review of reviews

## Abstract

**Objective:**

There are many systematic reviews of weight management interventions delivered by healthcare professionals (HCP), but it is not clear under what circumstances interventions are effective due to differences in review methodology. This review of systematic reviews synthesises the evidence about: (a) the effectiveness of HCP-delivered weight management interventions and (b) intervention and sample characteristics related to their effectiveness.

**Design:**

The review of reviews involved searching six databases (inception – October 2020). Reviews were included if they were (a) systematic, (b) weight management interventions delivered, at least partially, by HCP, (c) of randomised controlled trials and (d) written in English. Data regarding weight management outcomes (e.g. weight) and moderating factors were extracted. Secondary analyses were conducted using study-level data reported in each of the reviews.

**Setting:**

The review included studies that were delivered by HCP in any clinical or non-clinical setting.

**Participants:**

Not applicable.

**Results:**

Six systematic reviews were included (forty-six unique studies). First-level synthesis showed that weight management interventions delivered by HCP are effective. The second-level synthesis found that interventions are only successful for up to 6 months, are most effective for women, non-Caucasians and adults and are most effective if they have at least six sessions.

**Conclusions:**

As interventions are only successful for up to 6 months, they are not sufficient for achieving and maintaining a healthy weight.

Excess weight is linked to increased morbidity and mortality^(1,2)^, and weight management is improved through changes in dietary intake and/or changes in physical activity. Healthcare professionals (HCP) are well placed to offer weight management interventions, and interventions delivered by HCP may be more effective than interventions delivered by other means (e.g. trained interventionists, peers and commercial programmes)^(3)^. As such, there has been a proliferation in interventions delivered by HCP designed to target behaviours associated with weight management (a search of databases found 1 study in the 1980s, 18 studies in the 1990s, 66 studies in the 2000s and 142 studies in the 2010s). However, it is important to assess the effectiveness of interventions delivered by HCP as they have many demands on their time^(4,5)^.

HCP are well placed to offer weight management interventions for a number of reasons. First, HCP have the opportunity as people on average visit a general practitioner (GP) 6·9 times per year in developed countries^(6)^. Second, many countries mandate HCP to provide opportunistic behaviour change interventions (e.g. Making Every Contact Count in the UK)^(7)^ on health behaviours such as those that lead to obesity. Third, HCP are trusted sources of health information so may be able to persuade people to attempt weight loss^(3)^. Indeed, advice from HCP is a motivating factor in weight loss; among overweight/obese people (*n* 208) who had been advised to lose weight by a HCP, 89 % reported wanting to lose weight and 68 % were actually attempting to lose weight^(8)^. A more recent UK survey of obese patients reported that 30 % of patients found advice from a HCP was a motivating factor in weight loss^(9)^ and that 25 % of those actively trying to lose weight had sought advice from a GP or nurse and were highly likely to use GP resources^(9)^. Furthermore, a large survey found that patients welcomed behaviour change interventions from GP^(10,11)^.

However, the effectiveness of HCP-delivered interventions when compared to alternatives is mixed. Commercial weight loss programmes that are not delivered by healthcare professions can also be effective for weight loss. In one study, Weight watchers and Jenny Craig were more effective than controls and non-commercial programmes at 12-month follow-up^(12)^. Although there were mixed results when the commercial weight loss programmes were compared to non-commercial programmes delivered by HCP, Weight Watchers was more effective when compared to behavioural counselling delivered by primary care providers but not when delivered by psychologists^(12)^. Moreover, another review indicated that 57 % of those who start commercial weight loss programmes lose less than 5 % of their body weight suggesting that the weight loss from these programmes is not clinically beneficial^(13)^.

In addition to assessing overall effectiveness of HCP-delivered weight management interventions, it is also important to identify which features of interventions may contribute to their success or failure. These features include the specific HCP group who deliver the intervention, the setting and the content such as behaviour change techniques (BCT) (these are the active ingredients of interventions such as goal setting, self-monitoring or feedback). This will help in determining who should receive these interventions, who should deliver the interventions and what the intervention content should be in order to maximise their success.

Existing reviews of HCP-delivered weight management interventions are limited in assessing the effectiveness of the interventions and in identifying successful features due to their narrowness of scope and some methodological issues. They are limited by: (a) focusing on restricted samples (e.g. patients with type 2 diabetes^(14)^), (b) including studies with designs that are not as robust as randomised controlled trials (e.g. case-controlled studies^(15)^), (c) mixing delivery by non-healthcare and qualified HCP^(16)^ and (d) not being truly systematic (e.g. not following a search and data extraction strategy^(17)^). It is therefore difficult to assess from current reviews what is the effectiveness of HCP-delivered weight management interventions, for whom such interventions work best (e.g. people of particular ages, ethnicities or genders) and what is the optimum content (e.g. duration and intensity, which behavior change techniques are included).

The present research will synthesise the information from existing systematic reviews. Included systematic reviews will only comprise studies that report randomised controlled trials (with any comparison group) of HCP-delivered weight management interventions (i.e. focused on maintaining a healthy weight or losing excess weight), that measure a weight outcome, physical activity or nutritional behaviour and include participants regardless of demographics, weight status or medical condition. This systematic review of systematic reviews will determine: (a) the effectiveness of interventions delivered by HCP at managing people’s weight, (b) which intervention factors (e.g. BCT used) influence the effectiveness of these interventions and (c) what sample characteristics modify the effectiveness of these interventions.

## Method

This is a systematic review of systematic reviews with a primary level analysis reported at the review level (i.e. the findings reported by the reviews) and a secondary analysis reported at the study level (i.e. a new analysis, using vote counting, based on information reported in the reviews about the individual studies). This extra analysis was undertaken to enable us to more accurately answer our research questions, to check the conclusions reported in the reviews and to aggregate the evidence.

The review protocol was registered in PROSPERO: 42017059888 (http://www.crd.york.ac.uk/prospero/display_record.php?ID=CRD42017059888).

### Search strategy and selection criteria

Web of Science, CINAHL, PsycInfo, PubMed, Cochrane, SportDiscus and SCOPUS databases were searched in December 2018 and updated in October 2020 to identify systematic reviews and meta-analyses that assessed the effectiveness of interventions for weight management delivered by HCP for all dates from inception. The search strategy used four filters and MESH terms were used wherever possible. The first filter was for HCP (e.g. ‘health personnel’), the second filter captured interventions (e.g. ‘health promotion’ OR ‘health communication’ OR ‘health education’), the third filter was for weight management (e.g. ‘exercise’ OR ‘lifestyle’) and the fourth filter was for study type (e.g. ‘systematic review’ or ‘literature review’). Ascendancy (i.e. checking citations of included reviews) and descendancy techniques (i.e. checking reference sections of included reviews) were used to identify further reviews.

Reviews were included if they were (a) systematic (i.e. provided details of a systematic search strategy such as a database list, search terms, inclusion and exclusion criteria), (b) weight management interventions (i.e. with a focus on weight management, e.g. weight loss, weight maintenance, BMI, diet or physical activity) delivered, at least partially, by HCP, on any population (excluding babies not eating solid foods), (c) contained only randomised controlled trials (the comparison group could be usual care, measurement only control and alternative intervention) and (d) were written in English (due to time and resource constraints). Reviews that did not meet the above criteria, or were surgical or focused primarily on pharmacological support were excluded. See Supplementary Materials for full search strategy.

Three reviewers (TE, JG and CK) examined 36 % of titles and abstracts independently, resulting in 88·72 % agreement (527/594). Discrepancies regarding full texts were resolved through discussion until 100 % agreement was made regarding inclusion or exclusion.

### Data analysis

The data extraction forms were piloted (the completed forms are shown in Tables 1–4). Details of the reviews were extracted (date range of the literature included/searched, inclusion and exclusion criteria, quality of review, overlap of studies with other included reviews and reported quality of included studies): sample characteristics of included studies (age, ethnicity, gender, nationality and medical conditions), intervention characteristics of included studies (diet and/or physical activity targeted, BCT used (using the Behaviour Change Technique Taxonomy V1^(18)^), duration of interventions (up to 9 m; 9 m and over), intensity of interventions (number of sessions), tailored or not tailored, setting of intervention (clinical and non clinical), type of HCP (GP/physician, nurse, dietitian, unspecified and various), type of comparison group used (alternative intervention, usual care and delayed intervention/measurement only)) and a summary of results related to our research questions.

**Table 1:** Quality of systematic reviews

	PICO in RQ/inclusion criteria	Protocol*	Explanation of study design	Search strategy*	Duplicate screening	Duplicate data extraction	List of exclusions*	Description of included studies	Risk of bias (RoB) measured*	Sources of funding for studies	Appropriate statistical methods*	RoB in meta-analysis	RoB accounted for*	Explanation of heterogeneity	Publication bias in meta-analysis*	Conflict of interest reported	
Ball *et al*. (2013)^(23)^	Yes	No	No	Partial Yes	No	No	No	Partial Yes	No	Yes	N/A	N/A	Yes	Yes	N/A	No	Critically low
Mohammad & Ahmad (2016)^(14)^	No	No	No	No	No	No	No	No	No	No	N/A	N/A	No	Yes	N/A	No	Critically low
Moller *et al*. (2017)^(24)^	Yes	No	No	No	No	Yes	No	Yes	Yes	No	Yes	No	No	Yes	No	Yes	Critically low
Petit Francis *et al.* (2017)^(25)^	No	No	No	No	Yes	Yes	No	Yes	No	No	N/A	N/A	Yes	No	N/A	Yes	Critically low
Tsai & Wadden (2009)^(26)^	Yes	No	No	No	Yes	No	No	No	Partial Yes	Yes	N/A	N/A	No	Yes	N/A	Yes	Critically low
Wadden *et al*. (2014)^(27)^	Yes	No	No	No	Yes	No	No	Yes	No	No	N/A	N/A	Yes	Yes	N/A	Yes	Critically low

PICO, participant/intervention/control/outcome; RQ, research question.

* The absence of these are classed as critical flaws.

**Table 2:** Systematic review characteristics

	Review type	Literature dates	Inclusion criteria	No of studies (reporting weight/diet or physical activity outcomes)	Quality of review confidence in results	Overlap of studies	Risk of bias(% at low risk of bias)
Ball *et al*.(2013)^(23)^	Narrative	Not reported but literature included 1989–2008	Population: adults with lifestyle chronic diseases Intervention: nutrition care taken in general practitioner consultations Comparison: usual care or no care Outcomes: overall dietary intake, energy consumption, macro-nutrient intake, body weight, BMI, waist circumference, blood pressure and serum lipid levels Study design: randomised controlled trials (RCT) (with identical baseline and follow-up measurements)	9	Critically low	Total: 3 studies; 33·33 % Three studies; 33·33 % appear in Tsai & Wadden (2009)	Quality Criteria checklist Clear research Q • Low risk (9 studies; 100 %) P selection unbiased • Low risk (6 studies; 66·66 %) • Unclear (3 studies; 33·33 %) Comparable study groups • Low risk (5 studies; 55·55 %) • Unclear (4 studies; 44·44 %) Participant withdrawals described • Low risk (6 studies; 66·66 %) • High risk (2 studies; 22·22 %) • Unclear (1 study; 11·11 %) Blinding • Low risk (1 study; 11·11 %) • Unclear (8 studies; 88·89 %) Description of intervention protocol • Low risk (4 studies; 44·44 %) • High risk (2 studies; 22·22 %) • Unclear (3 studies; 33·33 %) Outcomes clearly defined • Low risk (7 studies; 77·77 %) • Unclear (2 studies; 22·22 %) Appropriate statistical analysis • Low risk (4 studies; 44·44 %) • Unclear (5 studies; 55·56 %) Conclusions supported by results • Low risk (8 studies; 88·88 %) • Unclear (1 study; 11·11 %) Unlikely funding bias • Low risk (9 studies; 100 %) Overall • Low risk (2 studies; 22·22 %) • Unclear (7 studies; 77·78 %)
Mohammad & Ahmad (2016)^(14)^	Narrative	Not specified	Population: adults with type 2 diabetes Intervention: weight loss interventions in primary care Comparison: usual care, other intervention Outcomes: Study design: not specified but only RCT in HCP analysis	2	Critically low	Total: 1 study; 50 % One study; 50 % appears in Wadden *et al*. (2014)	Not reported
Moller *et al*. (2017)^(24)^	Meta-analysis	Up to April 2017	Population: patients with type 2 diabetes Intervention: nutrition therapy Comparison: dietary advice Outcomes: BMI Study design: RCT	5	Critically low	Total: 0 studies; 0 %	Cochrane risk of bias tool Selection bias: Sequence generation • Low risk (3 studies; 60 %) • Unclear (1 study; 20 %) • High risk (1 study; 20 %) Selection bias: Allocation concealment • Low risk (2 studies; 40 %) • Unclear (3 studies; 60 %) Performance bias (blinding of participants and personnel) • Unclear (4 studies; 80 %) • High risk (1 study; 20 %) Detection bias (blinded outcome assessment) • Unclear (5 studies; 100 %) Attrition bias • Low risk (4 studies; 80 %) • High risk (1 study; 20 %) Reporting bias • Low risk (3 studies; 60 %) • Unclear (2 studies; 40 %) Other bias: • Low risk (4 studies; 80 %) • Unclear (1 study; 20 %)
Petit Francis *et al*. (2017)^(25)^	Narrative	2006–2016	Population: not specified Intervention: weight loss or weight loss maintenance interventions delivered by nurses Comparison: usual care, measurement only, other intervention and delayed intervention Outcomes: weight-related outcomes Study design: RCT	20	Critically low	Total: 0 studies; 0 %	Jadad scale (maximum 5): Randomisation • Not reported (20 studies; 100 %) Blinding • Not reported (20 studies; 100 %) An account of all patients • Not reported (20 studies; 100 %) Overall • Score of 1 (2 studies; 10 %) • Score of 2 (6 studies; 30 %) • Score of 3 (7 studies; 35 %) • Score of 4 (5 studies; 25 %)
Tsai & Wadden (2009)^(26)^	Narrative	1950–Jan 2009	Population: adults Intervention: counselling for weight loss conducted by primary care provider in the USA (or in a setting that simulated primary care in the USA) Comparison: placebo, usual care and alternative intervention Outcomes: weight loss Study design: RCT	10	Critically low	Total: 3 studies; 30 % 3 studies; 30 % appear in Ball *et al.* (2013)	Consort criteria/Agency for Healthcare Research/Quality Methods Guide for Comparative Effectiveness reviews Overall • Good quality (2 studies; 20 %) • Fair quality (8 studies; 80 %)
Wadden *et al*. (2014)^(27)^	Narrative	Jan 1980–June 2014	Population: overweight or obese adults recruited from primary care settings Intervention: behavioural weight loss counselling that included diet, physical activity and behavioural strategies, delivered by primary care practitioners or trained interventionists, for at least 3 months with at least 6 months follow-up and ≥15 participants per group Comparison: comparator intervention Outcomes: objectively measured change in weight Study design: RCT	4	Critically low	Total: 1 study; 25 % 1 study; 25 % appears in Mohammad & Ahmad (2016)	Obesity guidelines Overall • Low risk (4 studies; 100 %)

**Table 3:** Summary of sample characteristics

	Age	Ethnicity	Gender	Nationality	Medical conditions
Ball *et al.* (2013)^(23)^	49·09 years (mean from 4 studies; 44 %) Not reported (5 studies; 55·56 %)	Most African American (1 study; 11·11 %) 27·5 % Caucasian (2 studies; 22·22 %) Not reported (6 studies; 66·67 %)	37·94 % (4 studies; 44 %) Mixed gender % not specified (5 studies; 55·56 %)	USA (5 studies; 55·56 %) Italy (2 studies; 22·22 %) Netherlands (1 study; 11·11 %) Australia (1 study; 11·11 %)	Type II diabetes (1 study; 11·11 %) Hyperlipidaemia, hypertension or type II diabetes (1 study; 11·11 %) Hyperlipidaemia (1 study; 11·11 %) Hyperlipidaemia, hypertension or overweight BMI >= 30 (1 study; 11·11 %) Hypertension (1 study; 11·11 %) Not reported (4 studies; 44 %)
Mohammad & Ahmad (2016)^(14)^	51·9 years (mean from 1 study; 50 %) Not reported (1 study; 50 %)	38·5 % Caucasian (mean from 1 study; 50 %) Not reported (1 study; 50 %)	79·7 % female (1 study; 50 %) Not reported (50 %)	Not reported (2 studies; 100 %)	Not reported (2 studies; 100 %)
Moller *et al*. (2017)^(24)^	58·8 years (mean from 5 studies; 100 %)	Not reported (5 studies; 100 %)	55·2 % female (5 studies; 100 %)	UK (1 study; 20 %) Taiwan (1 study; 20 %) USA (1 study; 20 %) New Zealand (1 study; 20 %) China (1 study; 20 %)	Type II diabetes (5 studies; 100 %)
Petit Francis *et al*. (2017)^(25)^	47·91 years (mean from 11 studies; 55 %) 3–5 years range (1 study; 5 %) 8–12 years range (1 study; 5 %) 17–21 years range (1 study; 5 %) 55–60 years range (1 study; 5 %) 45+ years (1 study; 5 %) Not reported (4 studies; 20 %)	37·69 % Caucasian	52·22 % female (18 studies; 90 %) Not reported (2 studies; 10 %)	USA (6 studies; 30 %) Netherlands (4 studies; 20 %) Australia (3 studies; 15 %) Norway (1 study; 5 %) Finland (1 study; 5 %) Russia (1 study; 5 %) Sweden (1 study; 5 %) UK (1 study; 5 %) Turkey (1 study; 5 %) Taiwan (1 study; 5 %)	Not explicitly reported
Tsai & Wadden (2009)^(26)^	46·74 years (mean from 9 studies; 90 %) 40–69 years range (1 study; 10 %)	Mostly African American (1 study; 10 %) 28·86 % Caucasian (7 studies; 70 %) Not reported (2 studies; 20 %)	79·11 % female (9 studies; 90 %) Not reported (1 study; 10 %)	USA (10 studies; 100 %)	Hyperlipidaemia (1 study; 10 %) Hypertension (1 study; 10 %) Type II diabetes (1 study; 10 %) Not reported (7 studies; 70 %)
Wadden *et al.* (2014)^(27)^	49·53 years (mean from 4 studies; 100 %)	58·23 % Caucasian (4 studies; 100 %)	80·10 % female (4 studies; 100 %)	Not reported (4 studies; 100 %)	Overweight/obese (4 studies; 100 %) ≥2 metabolic syndrome conditions (1 study; 25 %)

**Table 4:** Summary of intervention characteristics

	Duration of follow-up	Behaviour change techniques used	Duration of interventions	Intensity of interventions	Tailored	Setting	Type of HCP	Control groups used
Ball *et al*. (2013)^(23)^	12 months (8 studies; 88·89 %) 18 months (1 study; 11·11 %)	Goal setting (1 study; 11·11 %) Review of goals (1 study; 11·11 %) Not reported (8 studies; 88·89 %)	6 months (1 study; 11·11 %) 9 months (1 study; 11·11 % 12 months (2 studies; 22·22 %) Not reported (5 studies; 55·56 %)	1 session/video (2 studies; 22·22 %) 1–3 sessions (1 study; 11·11 %) 4 sessions (1 study; 11·11 %) 6 sessions (2 studies; 22·22 %)	Tailored (4 studies; 44·44 %) Not tailored (1 study; 11·11 %) Not reported (4 studies; 44·44 %)	General practitioner (GP) practices (9 studies; 100 %)	GP (9 studies; 100 %)	Alternative intervention (3 studies; 33·33 %) Usual care (66·66 %)
Mohammad & Ahmad (2016)^(14)^	24 months (2 studies; 100 %)	Pharmacological support (1 arm of 1 study) Not reported (1 arm of 1 study; and 2 arms of 1 study)	6 months (1 study; 50 %) Not reported (1 study; 50 %)	Not reported (1 study; 50 %) 8 sessions (1 study; 50 %)	Not reported (2 studies; 100 %)	Primary care (2 studies; 100 %)	Primary care provider/physician (2 studies; 100 %)	Usual care (1 study; 50 %) Alternative intervention (1 study; 50 %)
Moller *et al*. (2017)^(24)^	12 months (5 studies; 100 %)	Self-monitoring (1 study; 20 %) Not reported (4 studies; 80 %)	6 months (2 studies; 40 %) 12 months (3 studies; 60 %)	1 session (1 study; 20 %) 3 sessions (1 study; 20 %) 4 sessions (2 studies; 40 %) 8 sessions (1 study; 20 %)	Tailored (1 study; 20 %) Not reported (4 studies; 80 %)	Not reported (5 studies; 100 %)	Dietitian (5 studies; 100 %)	Usual care (1 study; 20 %) Alternative intervention (4 studies; 80 %)
Petit Francis *et al.* (2017)^(25)^	3 months (1 study; 5 %) 4 months (3 studies; 15 %) 6 months (3 studies; 15 %) 7 months (1 study; 5 %) 12 months (3 studies; 15 %) 21 months (1 study; 5 %) 24 months (4 studies; 20 %) 30 months (1 study; 5 %) 36 months (1 study; 5 %) Not reported (2 studies; 10 %)	Goal setting (6 studies; 30 %) Self-monitoring (5 studies; 25 %) Feedback (3 studies; 15 %) Planning (2 studies; 10 %) Instruction (1 study; 5 %) Pharmacological support (1 study; 5 %) Not reported (10 studies; 50 %)	1–2 months (1 study; 5 %) 3 months (1 study; 5 %) 4 months (3 studies; 15 %) 6 months (4 studies; 20 %) 7 months (1 study; 5 %) 12 months (4 studies; 20 %) 24 months (1 study; 5 %) Not reported (5 studies; 25 %)	4 sessions (1 study; 5 %) 4–6 sessions (1 study; 5 %) 6 sessions (4 studies; 20 %) 10 + sessions (6 studies; 30 %) Not reported (8 studies; 40 %)	Tailored (3 studies; 15 %) Not reported (17 studies; 85 %)	Primary care (3 studies; 15 %) Homes (3 studies; 15 %) School (2 studies; 10 %) Child care facilities (1 study; 5 %) Vaccination centre (1 study; 5 %) Inpatient/community (1 study; 5 %) Outpatient/home (5 %) Work (1 study; 5 %) Community mental health (1 study; 5 %) GP practice (1 study; 5 %) Health centre (1 study; 5 %) Primary care/home (1 study; 5 %) Not reported (4 studies; 20 %)	Nurse (13 studies; 65 %) Nurse and physiotherapist (2 studies; 10 %) Nurse & dietitian (2 studies; 10 %) Nurse and physiologist (1 study; 5 %) Nurse and occupational therapist/dietitian (1 study; 5 %) Nurse, dietitian, physiotherapist and GP (1 study; 5 %)	Alternative intervention (10 studies; 50 %) Usual care (6 studies; 30 %) Measurement only/delayed intervention (4 studies; 20 %)
Tsai & Wadden (2009)^(26)^	6 months (1 study; 10 %) 12 months (6 studies; 60 %) 28 months (1 study; 10 %) 24 months (2 studies; 20 %)	Self-monitoring (4 studies; 40 %) Pharmacological support (3 studies; 30 %) Goal setting (2 studies; 20 %) Goal review (1 study; 10 %) Not reported (6 studies; 60 %)	6 months (1 study; 10 %) 9 months (1 study; 10 %) 12 months (5 studies; 50 %) 24 months (2 studies; 20 %) Not reported (1 study; 10 %)	1 session (1 study; 10 %) 4 sessions (1 study;10 %) 6 sessions (1 study; 10 %) 8 sessions (2 studies; 20 %) 10 + sessions (4 studies; 40 %) Not reported (1 study; 10 %)	Tailored (2 studies; 20 %) Not reported (8 studies; 80 %)	GP clinic (3 studies; 30 %) Primary care (1 study; 10 %) Research centre (1 study; 10 %) Not reported (5 studies; 50 %)	Primary care physicians/GP (7 studies; 70 %) Dietician (2 studies; 20 %) Physician, nurse and dietitian (1 study; 10 %)	Usual care (4 studies; 40 %) Placebo/alternative drug (2 studies; 20 %) Alternative intervention (3 studies; 30 %) Physician training only (1 study, 10 %)
Wadden *et al*. (2014)^(27)^	12 months (3 studies; 75 %) 24 months (1 study; 25 %)	Self-monitoring (2 studies; 50 %) Goal setting (1 study; 25 %) Pharmacological support (1 arm of one study) Not reported (1 study; 25 %)	6 months (2 studies; 50 %) 12 months (1 study; 25 %) Not reported (1 study; 25 %)	2 sessions (1 study; 25 %) 8 sessions (2 studies; 50 %) 10+ sessions (1 study; 25 %)	Tailored (1 study; 25 %) Not reported (3 studies; 75 %)	Primary care settings (4 studies; 100 %)	Primary care practitioners (4 studies; 100 %)	Usual care (3 studies; 75 %) Alternative intervention (1 study; 25 %)

HCP, healthcare professionals.

We also noted if weight loss was clinically significant. The reviews needed to report (a) the number of participants who lost at least 5 % of their initial body weight (which is enough to improve health and reduce weight-related complications^(19)^), (b) conduct the follow-up for up to 2 years (a loss of 2·5–5·5 kg at 2 years reduces the risk of developing diabetes by 30–60 %^(20)^) or (c) report a clinically significant outcome alongside the weight loss.

The systematic reviews often did not go beyond examining the main effects of interventions delivered by HCP to consider possible moderating effects (e.g. of sample and intervention characteristics). However, the reviews often reported this information at individual study level (e.g. mean age of sample for each study and profession of healthcare personnel who delivered the intervention) and so we extracted these data in order to conduct our own analyses to examine what constituted a ‘successful’ intervention. The planned meta-analysis could not go ahead as the reviews did not report the necessary information in the primary studies. Instead, we defined a ‘successful’ intervention as one in which there was either a statistically significant difference between intervention and control groups in BMI, weight loss or number of participants achieving at least 5 % weight loss at any time point. Studies that reported unusual thresholds for weight loss (e.g. number of participants losing at least 6 lbs) were classified as ‘unclear’. Thus, we were able to determine the likelihood of an intervention being successful with a range of potential moderators (e.g. age). If studies had multiple arms, each arm was compared to the control and treated as a separate study. If studies did not report an overall result but reported results separately for different samples, the different samples were treated as separate studies.

A vote counting analysis was used to compare categories within each moderator – these categories were split as follows. For study design, the length of follow-up was based on 6 months (as per the transtheoretical model definition of behavioural maintenance)^(21)^, 7–12 months and over 12 months to reflect the most common follow-ups reported in the reviewed studies.

For sample characteristics, gender was split into quartiles based on the percentage of females in the sample. Age was split, using mean age, into children and young adults (< 24 years), adults (24–43 years), middle-aged adults (44–64 years) and older adults (65 years) to match commonly used age categories (e.g. used in MeSH terms). Ethnicity was split into under 50 % non-Caucasian and greater than 50 % Caucasian. The nationality of the sample was broken down by continent as these groups were likely to have different cultures that affect weight loss and maintenance. Comorbidities were broken down into common ones (type 2 diabetes, hyperlipidaemia, hypertension, cardiac conditions and metabolic syndrome) and various types that were more than one comorbidity. Overweight and obese were split into under 50 % of the sample or over 50 % of the sample.

For intervention characteristics, the duration was split into up to 9 months and 12 months and over to reflect the range of durations in the studies included in the review. The number of sessions was split into under 6, 6–10 and over 10 to reflect the studies included in the review. Tailored was split into tailoring or no tailoring. Setting was split into clinical *v*. non-clinical as there were too few studies to report this to break this down further. Type of HCP was based on the reported HCP (GP/physician, nurse, dietitian, unspecified primary care provider or various HCP in multidisciplinary teams). The comparison group was broken down into the groups used in the studies (alternative intervention, usual care, delayed intervention/measurement only and placebo). Theory was broken down into theory used and theory not used. The BCT used were from the BCT reported in the studies (goal setting, goal review, pharmacological support, action planning, self-monitoring, instructions and feedback).

### Study quality assessment

The quality of the systematic reviews was assessed using the AMSTAR2 scoring system^(22)^. The AMSTAR2 system involves rating reviews (as yes, partial yes or no – the yes is a positive result) on if it included (1) a research question and inclusion criteria that included components of PICO (i.e. described participants, intervention, control and outcomes), (2) an explicit statement that the review methods were established prior to the review, for example, using a protocol/pre-registering, (3) an explanation of selection of study designs included in the review, (4) a comprehensive search strategy, (5) duplicate screening, (6) duplicate data extraction, (7) list of exclusions with justifications, (8) description of included studies, (9) measurement of risk of bias, (10) sources of funding for the studies included in the review, (11) appropriate statistical methods for meta-analysis, (12) potential impact of risk of bias on meta-analysis, (13) risk of bias accounted for when discussing the results, (14) exploration of heterogeneity, (15) publication bias in meta-analysis and (16) conflict of interest. Items 2, 4, 7, 9, 11, 13 and 15, if not included, are classed as critical flaws. The confidence in the results is rated as ‘High’ if there are no or only one non-critical weakness; ‘Moderate’ if they have more than one non-critical weakness, ‘Low’ if they one critical flaw (with or without non-critical weakness) and ‘Critically low’ if they have more than one critical flaw (with or without non-critical weakness).

## Results

The literature search, conducted in December 2018, identified 1782 references (no further references were found with an updated search in 2020 or ascendancy or descendancy searches). After duplicates were removed, 1632 were assessed for inclusion, of which 6 papers^(14,23–27)^, 5 without meta-analysis^(14,23,25–27)^ and 1 with meta-analysis^(24)^, met our inclusion criteria (see online Supplemental Materials Fig. 1). Most of the reviews focused on weight loss only (3/6; 50 %), with two reviews not mentioning weight loss nor management (2/6; 33 %) and one review mentioning both weight loss and weight management (1/6; 17 %).

The quality of the systematic reviews was assessed by the AMSTAR2^(22)^. All of the reviews had more than one critical flaw: none of them had a protocol or a list of excluded studies with reasons; only one had a search strategy (and that was partial and not comprehensive); only one had measured risk of bias and one had done this partially; and three had accounted for the risk of bias in the discussion. Although the meta-analysis did use appropriate statistical methods and explored publication bias, it had some of the other aforementioned critical flaws. Therefore, all the reviews were categorised as critically low confidence in the results (see Table 1 for quality of systematic reviews).

The reviews report findings from fifty studies (forty-six of these are unique studies with four studies that are included in more than one review). Review authors searched the databases from inception to 2017, but the included studies were published between 1989 and 2017. The reviews included between two and twenty papers (see Table 2 for a summary of review characteristics). The forty-six studies were conducted mainly in the USA (24; 52·17 %), with twelve in Western Europe (26·09 %), five in Australia/New Zealand (10·87 %), three in Asia (6·52 %) and two in Eastern Europe (4·35 %). The risk of bias for each included study varied from low to high (see Table 2). The samples were mainly adult, mainly female, but they varied in ethnicity and presence of medical conditions (see Table 3 for summary of sample characteristics).

The duration of the interventions varied from 1 to 24 months, varied in intensity from 1 to 10 plus sessions, were delivered in a variety of settings including clinical and non-clinical settings and were delivered by various HCP but mainly by GP/physicians; most studies did not report BCT (those that did mostly reported using goal setting and/or self-monitoring but also included feedback, review of goals, pharmacological support, planning and instruction on how to perform the behaviour). The comparison groups used were mainly alternative interventions or usual care; the follow-ups varied from 3 months to 36 months (see Table 4 for a summary of intervention characteristics).

### Interventions for weight loss or maintenance

The systematic reviews suggested that interventions delivered by HCP were effective for weight loss or BMI reduction^(14,23,24,26,27)^, waist circumference reduction^(24)^, and dietary intake improvements^(23)^, and that the weight loss was clinically significant^(14,24,27)^ (although one systematic review reported that few studies were clinically significant^(26)^). The interventions included GP/primary care physicians^(14,23)^ and dietitian-delivered interventions^(24)^ for adults who had a chronic disease, nurse-delivered interventions for children and adults^(24)^ and primary care physician-delivered interventions for adults^(26,27)^.

Weight loss or BMI reduction in adults with chronic diseases was reported as between −0·40 and −2·30 kg/m^2^ and −0·20 to −0·81 kg/m^2^ for the most recent five studies reviewed for interventions delivered by GP (clinical significance was not reported^(23)^). The review also reported that 55 % (5/9) of studies improved dietary intake^(23)^. Five interventions delivered by dietitians for people with type 2 diabetes resulted in weight loss or BMI reduction of −2·10 kg and −0·55 kg/m^2^ and also a significant reduction in HbA1c^(24)^. The review also found that the two studies that measured waist circumference (2/2; 100 %) reported decreases of between –1·60 and –2·80 cm^(24)^. A further systematic review of interventions conducted in a primary care setting for adults with diabetes reported that all four comparisons (100 %) were statistically and clinically significant, although exact figures were not provided^(14)^. Weight loss from primary care provider interventions for adults ranged from –0.10 to –2·30 kg and with additional pharmacological support, –1·70 to –7·50 kg (only one of the four, 25 %, was clinically significant^(26)^). Overweight and obese adults receiving interventions by primary care providers found that all four studies (4/4; 100 %) reported either statistically or clinically significant weight loss^(27)^; a further review found the same results (2/2; 100 %) but did not report actual weight loss figures^(14)^. A systematic review of interventions conducted on adults and children by nurses reported 65 % (13/20) of studies resulted in significant weight loss or BMI reduction, although seven studies reported null effects (7/20; 35 %) on all outcome measures (clinical significance of weight loss was not reported)^(25)^.

No systematic reviews explored effectiveness over time as a moderator. Our analysis of individual studies found that interventions were likely to be successful for up to 6 months (76·92 % of interventions successful; 13/20) with success rates falling at 7–12 months (41·38 % of interventions successful; 12/29) and over (42·86 % of interventions successful; 6/14) (see Supplemental Materials Table 1 available online).

Only one systematic review considered the potential moderating effect of sample characteristics: males may benefit slightly more than females from nurse-delivered interventions, and there were no differences due to age or nationality^(25)^. Our synthesis found that HCP-delivered weight management interventions seem to be more successful in reducing weight loss or BMI when the sample was predominantly female (if over 75 % of sample were female, 91·67 % of interventions were successful; 11/12), predominantly non-Caucasian (83·33 % of interventions were successful if non-Caucasians made up > 50 % of sample), have a mean age of between 24 and 43 years (83·33 % of interventions successful; 16/28) and the sample was non-European (50 % of USA, New Zealand/Australian and Asian studies successful; 19/38). The systematic reviews did not look at medical conditions or weight status as moderators. There were too few studies to explore medical conditions as a moderator (see Supplemental Materials Table 2 – available online).

Systematic reviews reported conflicting results with respect to intervention intensity^(23,26)^ but found that nurses in multidisciplinary teams were most effective^(25)^ as were interventions that included pharmacological support^(26)^). Reviews did not explore duration of interventions, setting or type of control group. Our synthesis showed that successful interventions were more likely to have over six sessions (over 60 % of interventions with six plus sessions were successful; 14/23), to use single healthcare professions (between 47·06 and 60·00 % were successful), to deliver the interventions rather than multidisciplinary teams (37·50 % of interventions were successful; 3/8), to report use of specific BCT (e.g. pharmacological support, self-monitoring, feedback, action planning; between 62·50 and 100 % of success rate) and to use a measurement only or delayed intervention control group (60 % interventions were successful; 3/5). Delivering the intervention in a clinical setting and increasing the duration past 6 months did not add notable benefit (see Supplemental Materials Table 3 – available online).

With regard to quality, only two systematic reviews examined the quality and significance of findings and found either no differences due to quality^(25)^, or that studies with a positive quality rating had significant positive effects on outcomes^(23)^. Our own analysis revealed no clear trends in the likelihood of successful weight loss or BMI reduction due to the quality of the study (see Supplemental Materials Table 4 – available online).

## Discussion

The six systematic reviews provided mixed results regarding the effectiveness of HCP-delivered interventions for weight loss. However, the confidence in these findings was rated as critically low. Regarding interventions for weight loss and/or weight maintenance, most of the systematic reviews suggested that HCP-delivered interventions were effective for actual weight loss (between – 0 10 kg and –7·50 kg) and clinically significant weight loss. Our own synthesis of forty studies from six systematic reviews found that the HCP-delivered interventions for weight loss were successful but only in the short term up to 6 months, after which the success rate dropped off, so only just under 42 % of included studies showed the interventions led to sustained weight loss after this time. Given that most interventions lasted for 6 months or more, this suggests that HCP-delivered interventions may not be successful for attaining and maintaining a healthy weight in the long term. This does not seem to be related to the duration of the intervention as this review showed no benefit of extending the interventions over 6 months. The problem may be that interventions do not include a sufficient number or the most effective BCT needed to encourage behavioural maintenance. A review found that the number of BCT was positively correlated with the effectiveness of interventions for overweight and obese adults^(28)^. The most successful behaviour change techniques for long-term maintenance (over 12 months) were goal setting, self-monitoring, feedback, graded tasks and adding objects to the environment^(28)^.

The single systematic review that looked at sample characteristics concluded that HCP-led interventions were slightly more effective for males, and there were no differences due to age or nationality^(25)^. Our own synthesis of forty studies from six systematic reviews contradicts this: HCP-delivered interventions were most likely to be effective in samples with a higher percentage of females, among adults (aged 24–43 years), with a greater percentage of non-Caucasian participants and were in non-European samples. It seems unlikely to be the case that the above groups have more successful weight management as they are less likely to be obese and have less complex needs than male, older aged European and Caucasian participants; there is no clear data that suggest this^(29,30)^. This suggests that HCP-delivered interventions may not be suitable for all target groups.

One systematic review reported that interventions were more effective if delivered by multidisciplinary teams. Our analysis found, in contrast, that the most effective interventions were delivered with only one type of HCP. It could be argued that this is because single healthcare professions usually deliver interventions to those people with less complex needs, and teams of mixed HCP are more likely to be used in people with more complex needs.

The reviews had mixed results regarding intensity, but our secondary analysis found that interventions were more successful if they were conducted over six or more sessions. This suggests that more intense intervention delivery is needed, thus potentially adding to the expense of HCP-delivered interventions for weight management.

Both systematic reviews and our analysis agreed that the use of pharmacological support increased effectiveness; and our analysis also reported the use of other specific BCT was related to effectiveness. The BCT listed included feedback, self-monitoring and action planning. This could be because studies that include details of BCT when describing interventions are more likely to be designed using theory and evidence from behavioural science. The success of these interventions is not surprising as using evidence-based techniques improves the effectiveness of interventions.

This systematic review of systematic reviews was limited as the reviews were rated as critically low in the quality assessment. There was limited reporting of outcomes such as number of people who lost 5 % of initial weight, so it was difficult to determine the clinical effectiveness of the interventions. There was also limited reporting of intervention details such as BCT used. A further limitation is that it was difficult, using the conclusions drawn from the included systematic reviews, to determine if weight loss from HCP-delivered interventions is greater than in those interventions delivered by non-HCP (e.g. commercial weight loss interventions) as the systematic reviews did not always: (a) provide an overall effect size, (b) report the actual differences in weight or BMI between control and intervention groups in the individual studies or (c) include studies that compared similar weight management interventions delivered by a HCP *v*. a non-HCP. To allow a comprehensive assessment of the effectiveness of HCP-delivered interventions, a systematic review is needed that: (a) quantifies the effect of HCP-delivered interventions through meta-analysis, (b) is broad in scope (i.e. to include all HCP and samples) and (c) explores the moderating effect of sample and intervention characteristics to inform best practice for HCP-delivered interventions.

This systematic review of systematic reviews suggests that HCP-delivered interventions can be effective for weight loss for up to 6 months but after this time, the effect is substantially reduced so the interventions may not be successful for attaining and maintaining a healthy weight. HCP-delivered weight interventions may be time-intensive as our findings suggest they should be delivered for six or more sessions. HCP-delivered weight loss interventions may not be effective for all target groups.

## Supplementary material

For supplementary material accompanying this paper visit https://doi.org/10.1017/S1368980021004481. click here to view supplementary material
