# Supplementary material for: Are interventions delivered by healthcare professionals effective for weight management? A systematic review of systematic reviews
Source: Public Health Nutr. 2021 Oct 28;25(4):1071–83. doi: 10.1017/S1368980021004481 (PMC9991715; doi:10.1017/S1368980021004481)
Supplement: Supplementary file 1 [file S1368980021004481sup.zip › S1368980021004481sup002.docx]

Supplementary materials

**PubMed Search**

Using mesh terms

Population

Health personnel

Intervention

Health promotion OR

Health communication OR

Health education

Outcome

Exercise

Life Style

**Web of Science Search**

**TOPIC:** (“Health professional” OR “Health practitioner” OR “Health personnel” OR Anaesthetist OR Audiologist OR Chiropractor OR Dentist OR Dietician OR Dietitian OR “General practitioner” OR Doctor Gynaecologist OR “Health visitor” OR Midwife OR Nurse OR Obstetrician OR Ophthalmologist OR Optician OR Osteopath OR Paediatrician OR Pathologist OR Pharmacist OR Psychiatrist OR Psychologist OR Radiologist OR “Social Worker” OR “Speech therapist” OR “Language therapist” OR Surgeon OR Physician OR “Primary care” OR “Secondary care”) *AND* **TOPIC:** ("Health Information" OR "Health Advice" OR "Health Intervention" OR "Service provision" OR "Behavior change" OR "behaviour change" OR "lifestyle intervention" OR Counselling OR "Health Communication" OR "Health Promotion" OR "Health education") *AND* **TOPIC:** (Diet OR Nutrition OR Fruit OR Vegetables OR Sugar OR Fat OR Fibre OR Salt OR “Physical activity” OR Exercise OR Inactivity OR “Weight loss” OR “weight management” OR BMI OR “Body mass index” OR “Sedentary behavior” OR "Sedentary Behaviour")

Filter by review

**CINAHL**

Using mesh terms

Population

Health personnel

Intervention

Health promotion OR

Health communication OR

Health education

Outcome

Exercise

Life Style

Publication type

Systematic review

Literature review

**PsycInfo**

(("Health professional" or "Health practitioner" or "Health personnel" or Anaesthetist or Audiologist or Chiropractor or Dentist or Dietician or Dietitian or "General practitioner" or Doctor or Gynaecologist or "Health visitor" or Midwife or Nurse or Obstetrician or Ophthalmologist or Optician or Osteopath or Paediatrician or Pathologist or Pharmacist or Psychiatrist or Psychologist or Radiologist or "Social Worker" or "Speech therapist" or "Language therapist" or Surgeon or Physician or "Primary care" or "Secondary care" or pediatrician) and ("Health Information" or "Health Advice" or "Health Intervention" or "Service provision" or "Behav* change" or "lifestyle intervention" or Counselling or Counseling or "Health Communication" or "Health Promotion" or "Health education")).id. and (Diet or Nutrition or Fruit or Vegetables or Sugar or Fat or Fibre or Salt or "Physical activity" or Exercise or Inactivity or "Weight loss" or “weight management” or BMI or "Body mass index" or "Sedentary behav*").af. and (review or "meta-analysis" or synthesis).ab.

**Cochrane**

Using mesh terms

Population

Health personnel

Intervention

Health promotion OR

Health communication OR

Health education

Outcome

Exercise

Life Style

**sportdiscus**

( “Health professional” OR “Health practitioner” OR “Health personnel” OR Anaesthetist OR Audiologist OR Chiropractor OR Dentist OR Dietician OR Dietitian OR “General practitioner” OR Doctor OR Gynaecologist OR “Health visitor” OR Midwife OR Nurse OR Obstetrician OR Ophthalmologist OR Optician OR Osteopath OR Paediatrician OR Pathologist OR Pharmacist OR Psychiatrist OR Psychologist OR Radiologist OR “Social Worker” OR “Speech therapist” OR “Language therapist” OR Surgeon OR Physician OR “Primary care” OR “Secondary care” OR pediatrician ) AND ( “Health Information” OR “Health Advice” OR “Health Intervention” OR “Service provision” OR “Behavio?r change” OR “lifestyle intervention” OR Counsel?ing OR “Health Communication” OR “Health Promotion” OR “Health education” ) AND ( Diet OR Nutrition OR Fruit OR Vegetables OR Sugar OR Fat OR Fibre OR Salt OR “Physical activity” OR Exercise OR Inactivity OR “Weight loss” OR “weight management” OR BMI OR “Body mass index” OR “Sedentary behavio?r” ) AND AB ( Review OR Meta-analysis OR synthesis )

refine results to academic articles only, literature reviews and meta analyses and english

Scopus

( KEY ( {Health professional}  OR  {Health practitioner}  OR  {Health personnel}  OR  anaesthetist  OR  audiologist  OR  chiropractor  OR  dentist  OR  dietician  OR  dietitian  OR  {General practitioner}  OR  doctor  OR  gynaecologist  OR  {Health visitor}  OR  midwife  OR  nurse  OR  obstetrician  OR  ophthalmologist  OR  optician  OR  osteopath  OR  paediatrician  OR  pathologist  OR  pharmacist  OR  psychiatrist  OR  psychologist  OR  radiologist  OR  "Social Worker"  OR  "Speech therapist"  OR  "Language therapist"  OR  surgeon  OR  physician  OR  "Primary care"  OR  "Secondary care"  OR  pediatrician )  AND  KEY ( {Health Information}  OR  {Health Advice}  OR  {Health Intervention}  OR  {Service provision}  OR  {Behavior change}  OR  {behaviour change}  OR  {lifestyle intervention}  OR  counselling  OR  counseling  OR  {Health Communication}  OR  {Health Promotion} )  AND  KEY ( diet  OR  nutrition  OR  fruit  OR  vegetables  OR  sugar  OR  fat  OR  fibre  OR  salt  OR  {Physical activity}  OR  exercise  OR  inactivity  OR  {Weight loss}  OR  bmi  OR  {Body mass index}  OR  {sedentary}  OR  {Weight management} )  0 )  AND  ( LIMIT-TO ( DOCTYPE ,  "re" ) )  AND  ( LIMIT-TO ( LANGUAGE ,  "English" ) )  AND  ( LIMIT-TO ( SRCTYPE ,  "j" ) )

Figure 1: Flow of papers through review

Records identified through database searching
(*n*=1782)

## Included

## Identification

Records after duplicates removed
(*n*=1632)

## Screening

Records excluded
(*n* = 1491)

Records screened
(*n*=1632)

Full-text articles excluded, with reasons
(*n*=135)

- Not a review of weight, diet or PA (*n*=21)
- Incudes studies other than those delivered by health care professionals (*n*=49)
- Includes studies other than randomized controlled trials (*n*=17)
- Not focused on weight management or dietary / PA behaviour (*n*=26)
- Is not systematic (*n*=19)
- Unavailable as full paper (*n*=1)
- Not in English (*n*=1)
- Did not report weight, diet or PA outcomes (*n*=1)

## Eligibility

Full-text articles assessed for eligibility
(*n*=141)

Papers included in review (*n*=6)

Supplementary Table 1

*Effectiveness of Intervention by Length of Follow Up for Weight Loss / Maintenance Studies*

| **Moderator** | **Significant WL (on at least one measure), BMI or dietary behaviour (on at least one measure)** | **Non significant** | **Unclear** |
| --- | --- | --- | --- |
| Time of follow up |  |  |  |
| Up to 6 months  WL and BMI  76.92% | 3M & 6M (Brown et al., 2011) WL at 3M but not at 6M  3M (Wang et al., 2012) WL  3M (Anderson et al., 2006) BMI  4M (Wadden et al., 2005) WL  4M (Gallagher et al., 2012) BMI & WL  6M (Ely et al., 2008) WL  6M (Martin et al., 2008) WL  6M (Wadden et al., 2011 – brief lifestyle) WL  6M (Tsai et al., 2010) WL  6M (Leemrijse et al., 2016) BMI |  | 6M (Logue et al., 2005) unclear if WL sig  6M (Andryukhin et al., 2010) BMI sig but measured as no in each cond with positive changes  6M (van der Veen et al., 2002) unclear if WL & BMI sig at 6m |
| 7-12 months  WL and BMI  41.38% | 7M (Alkon et al., 2014) BMI  9M & 12M (Martin et al., 2008) WL at 9M but not at 12M  12M (Ashley et al., 2001) WL  12M (Christian et al., 2011) WL & 5% BODY WEIGHT  12M (Kumanyika et al., 2012) number with 5% BODY WEIGHT at 12m but WL non sig  12M (Ockene et al., 1999 – physician training + office support) WL  12M (Poston et al., 2006) WL  12M (ter Bogt et al., 1999; 2011) WL  12M (Wright et al., 2013 – girls) BMI  12M (Sacerdote et al., 2006) BMI but not WL  12M (Andrews et al., 2011) WL and BMI  12M (Coppell et al., 2010) BMI but not WL | 12M (Alli et al., 1992) WL  12M (Cohen et al. 1991) WL  12M (Tsai et al., 2010) WL  12M (Wadden et al., 2005) WL  12M (Brown et al., 2014) WL  12M (Fulkerson et al., 2015) BMI  12M (Wright et al., 2013 – boys) BMI  12M (Salkeld et al., 1997 – video) WL & BMI  12M (Salkeld et al., 1997 – video + self help) WL & BMI  12M (Wilson et al., 2016) BMI & WL  12M (Beresford et al., 1997) WL & BMI  12M (van der Veen et al., 2002) WL & BMI  12M (Ockene et al., 1999 – physician training) WL  12M (Huang et al., 2010) BMI  12M (Franz et al., 1995) WL and BMI | 12M (Christian et al.; 2008) WL sig but measure was lost 6lbs or more; BMI, and WC non sig  12M (Logsdon et al., 1998) WL sig but measure was no who lost 5lbs or more |
| 12+ months  WL & BMI  42.86% | 24M (Appel et al., 2011 – in person support) WL & 5% BODY WEIGHT  24M (Appel et al., 2011 – remote support) WL & 5% BODY WEIGHT  24M (Hauptman et al., 2000) WL  24M (Wadden et al., 2011 – enhanced lifestyle) WL but unclear if 5% BODY WEIGHT sig  24M (Drevenhorn et al., 2012) BMI  24M (Lemon et al., 2014) WL & BMI | 18M (Martin et al., 2008) WL  24M (Logue et al., 2005) WL  24M (Wadden et al., 2011 – brief lifestyle) 5% BODY WEIGHT  24M (Hesselink et al., 2015) WL & BMI  24M (Fulkerson et al., 2015) BMI  24M (Kokkvoll et al., 2015) BMI  36M (ter Bogt et al., 2009; 2011) WL  30M (Vermunt et al., 201; 2012) WL & BMI |  |
| 7-12 months  DIET BEHAVIOUR  44.00% | 12M (Ockene et al., 1999 – physician training + office support) FAT but not FIBRE  12M (Beresford et al., 1997) FAT & FIBRE  12M (van der Veen et al., 2002) FAT but not clear if ENERGY INTAKE sig  12M (Sacerdote et al., 2006) FRUIT & VEG, OLIVE OIL, FISH & FAT | 12M (Logsdon et al., 1998) DIETARY INTAKE  12M (Christian et al.; 2008) DIETARY INTAKE  12M (Ockene et al., 1999 – physician training) FIBRE & FAT  12M (Salkeld et al., 1997 – video) FAT  12M (Salkeld et al., 1997 – video + self help) FAT |  |
| 12+ months  DIET BEHAVIOUR |  | 18M (Martin et al., 2008) DIETARY INTAKE |  |
| Unspecified time frame  20% | (Hacihasaoglu et al., 2011 – medication compliance + education) BMI | (Valve et al., 2013) BMI  (Goodman et al. 2008) BMI  (Hacihasaoglu et al., 2011 – medication adherence and questions) BMI  (Usher et al., 2013) WL & BMI |  |

Supplementary Table 2

*Effectiveness of Intervention by Sample Characteristics for Weight Loss / Maintenance Studies*

| **Moderator** | **Significant**  **In one WL or BMI measure at any time point** | **Non significant**  **On any WL measure or BMI at any time point** | **Unclear** |
| --- | --- | --- | --- |
| Gender |  |  |  |
| Sample with < 25% females  25% | 19% (Leemrijse et al., 2016) BMI at 6m | 0% (Wright et al., 2013 - boys) BMI at 12m (boys only)  5.9% (Wilson et al., 2016) WL and BMI at 12m  18.7% (Goodman et al., 2008) BMI at non specified time |  |
| Sample with 25% to 50% females  44.44% | 35% (Andrews et al., 2011) WL and BMI at 12m  41% (Gallagher et al., 2012) WL and BMI at 4m  45% (Brown et al., 2011; 2014) WL at 3m, NS at 6m & 12m  46% (Alkon et al., 2014) BMI at 7m | 46.7% (Hesselink et al., 2015) WL and BMI at 24m  47% (Usher et al. 2013) WL and BMI at unspecified time  48% (Fulkerson et al., 2015) BMI at 12m and 24m  48.74% (Salkeld et al., 1997 – video intervention) WL & BMI at 12m  48.74% (Salkeld et al., 1997 – video intervention + self help) WL & BMI at 12m |  |
| Sample with 51% to 75% females  43.75% | 52% (Hacihasaoglu et al., 2011 – med compliance + education) BMI at unspecified time  52% (ter Bogt et al., 2009; 2011) WL at 12m but ns at 36m  56% (Ockene et al., 1999 – physician training + office support) – WL at 12m  59% (Coppell et al., 2010) BMI at 12m but not WL  67% (Lemon et al., 2014) WL and BMI at 24m  68.3% (Wang et al., 2012) WL at 3m  72% (Hauptman et al., 2000) WL at 24m | 52% (Hacihasaoglo et al., 2011 – med adherence + questions) BMI at unspecified time  54% (Kokkvoll et al., 2015) BMI at 24m  55% (Franz et al., 1995) BMI and WL at 12m  56% (Ockene et al., 1999 – physician training only) WL at 12m  66% (Huang et al.,2010) BMI at 12m  68.4% (Christian et al., 2011) WL at 12m  68% (Logue et al., 2005) unclear if sig WL at 6m, non sig at 12m | 69.4% (Andryukhin et al., 2010) BMI sig at 6m but measured as no in each cond with positive changes  67% (Christian et al., 2008) - non sig actual weight loss / BMI at 12m but more had over 6lbs weight loss in intervention than comparison group |
| Samples with over 75% females  91.67% | 77% (Ely et al., 2008) WL at 6m  79.7% (Wadden et al., 2011 – brief lifestyle) WL sig at 6m but not at 24m  79.7% (Wadden et al., 2011 – enhanced lifestyle) WL sig at 24m  80% (Wadden et al., 2005) WL at 4m but NS at 12m  84.3% (Kumanyika et al., 2012) number with 5% WL at 12m but WL non sig  88% (Tsai et al., 2010) WL at 6m but not at 12m  92% (Poston et al., 2006) WL at 12m  100% (Martin et al., 2008) WL at 6m and 9m but not at 18m  100% (Wright et al., 2013 - girls) BMI at 12m  100% (Anderson et al., 2006) BMI at 3m  100% (Ashley et al., 2001) WL at 12 | 100% (Valve et al., 2013) BMI @ unspecified time |  |
| Age | | | |
| Under 24 years  33.33% | 3-5 years (Alkon et al., 2014) BMI at 7m  8 – 12 years (Wright et al., 2013 - girls) BMI at 12m | 10.25 (Kokkvoll et al., 2015) BMI at 24m  10.3 (Fulkerson et al., 2015) BMI at 12m  8 – 12 years (Wright et al., 2013 - boys) BMI at 12m  19 median (17 – 21 range) (Valve et al., 2013) BMI at non specified time |  |
| 24 - 43 years  83.33% | 40.4 (Ashley et al., 2001) WL at 12m  41 (Poston et al., 2006) WL at 12m  41.7 (Martin et al. 2008) WL at 6m but not 12m and 18m  42.5 (Hauptman et al., 2000) WL at 24m  43.6 (Wadden et al., 2005) WL at 4m but not at 12m | 40-69 (Logue et al., 2005) WL unclear at 6m and non sig at 24m |  |
| 44 - 65 years  57% | 44.65 (Brown et al., 2011; 2014) WL at 3m but not at 6m and 12m  46.7 (Wang et al., 2012) WL at 3m  47.2 (Kumanyika et al., 2012) WL no with > 5% WL at 12m but not actual WL  49.3 (Ockene et al., 1999 – physician training + office support) WL at 12m  49.4 (Tsai et al., 2010) WL at 6m but not at 12m  49.5 (Ely et al., 2008) WL at 6m  49.6 (Christian et al., 2011) WL at 12m  51.9 (Wadden et al., 2011 – brief lifestyle) WL at 6m but not at 24m  51.9 (Wadden et al., 2011 – enhanced lifestyle) WL at 24m  56 (ter Bogt et al., 2009; 2011) WL at 12m but not at 36m  56.8 (Hacihasaoglu et al., 2011 – med compliance + education) BMI at unspecified time  58 (Coppell et al., 2010) BMI but not WL at 12m  55-60 years (Anderson et al., 2006) BMI at 3m  60 (Andrews et al., 2011) BMI and WL at 12m  60.4 (Leemrijse et al., 2016) BMI at 6m  63.59 (Gallagher et al., 2012) WL and BMI at 4m | 40-69 (Logue et al., 2005) WL unclear at 6m and non sig at 24m  45+ (Hesselink et al., 2015) WL and BMI at 24m  46.5 (Wilson et al, 2016) WL and BMI at 12m  49.3 (Ockene et al., 1999 – physician training only) WL at 12m  52.17 (Salkeld et al., 1997 – video) WL and BMI at 12m  52.17 (Salkeld et al., 1997 – video + self help) WL and BMI at 12m  56 (Franz et al., 1995) WL and BMI at 12m  56.8 (Hacihasaoglu et al., 2011 – med adherence + questions) BMI at unspecified time  57 (Huang et al., 2010) BMI at 12m  59.5 (Cohen et al., 1991) WL at 12m  64.8 (Goodman et al., 2008) BMI at unspecified time | 53.2 (Christian et al., 2008) WL sig at 12m (but not actual WL only no who lost 6lbs only), BMI ns at 12m |
| 65 and over  0% |  |  | 67 (Andryukhin et al., 2006) BMI sig at 6m but measured as no in each cond with positive changes |
| Ethnicity (% who are non-Caucasian) | | | |
| Up to 50% non-Caucasian  62.50% | 4.1% (Lemon et al., 2014) WL and BMI at 24m  5% non-white (Ockene et al., 1999 – physician training + office support) WL at 12m  11% (Hauptman et al., 2000) WL at 24m  13% (Ely et al., 2008) WL at 6m  21% (Gallagher et al., 2012) WL and BMi at 4m  32% (Wadden et al., 2005) WL at 4m but not at 12m  38.5% black (Wadden et al., 2011 – brief lifestyle) WL at 6m but not at 24m  38.5% black (Wadden et al., 2011 – enhanced lifestyle) WL at 24m  40.5% (Brown et al., 2011; 2014) WL at 3m but ns at 6m and 12m  49.4% (Christian et al., 2011) WL at 12m | 5% non white (Ockene et al., 1999 – physician training only) WL at 12m  23.9% (Wilson et al., 2016) WL and BMI at 12m  27% (Logue et al., 2005) WL unclear at 6m non sig at 14m  29% (Fulkerson et al., 2015) BMI at 12m  29% (Usher et al., 2013) WL and BMI at unspecified time | 50% Latino (Christian et al., 2008) sig at 12m for no with over 6lbs but not actual WL |
| 51% and above non-Caucasian  83.33% | 54% (Alkon et al., 2014) WL at 7m  64% (Poston et al., 2006) WL at 12m  65% (Kumanyika et al., 2012) no with > 5% WL sig at 12m but not actual WL  80% (Tsai et al., 2010) WL sig at 6m but non sig at 12m  100% (Wright et al., 2013 – girls) BMI at 12m | 100% (Wright et al., 2013 – boys) BMI at 12m |  |
| Nationality |  |  |  |
| Western Europe  41.67% | Italy (Sacerdote et al., 2006) WL non sig at 12m; BMi sig at 12m  Sweden (Drevenhorn et al., 2012) BMI at 24m  Netherlands (Leemrijse et al., 2016) BMI at 6m  Netherlands (ter Bogt et al., 2009; 2011) WL at 12m but not at 36m  UK (Andrews et al., 2011) BMI and WL at 12m | Italy (Alli et al., 1992) WL at 12m  Norway (Kokkvoll et al., 2015) BMI at 24m  Finland (Valve et al., 2013) BMI at unspecified time  UK (Goodman et al., 2008) BMI at unspecified time  Netherlands (Hesselink et al., 2015) WL and BMI at 24m  Netherlands (Van der Veen et al., 2002) unclear if WL & BMI sig at 6m, WL & BMI ns at 12m  Netherlands (Vermunt et al., 2011; 2012) WL and BMI at 30m |  |
| Eastern Europe  33.33% | Turkey (Hacihasaoglu et al., 2011 – med compliance and education) BMI at unspecified time | Turkey (Hacihasaoglu et al., 2011 – med adherence + questions) BMI at unspecified time | Russia (Andryukhin et al., 2010) BMI sig at 6m but measured as no in each cond with positive changes |
| North America  50% | USA (Martin et al., 2008) WL at 6m and 9m but not at 12 and 18m  USA (Ockene et al., 1999 – physician training + office support) WL at 12m  USA (Alkon et al., 2014) BMI at 7m  USA (Wright et al., 2013 – girls) BMI at 12m  USA (Brown et al., 2011; 2014) WL at 3m but ns at 6 and 12m  USA (Lemon et al., 2014) WL and BMI at 24m  USA (Hauptman et al., 2000) WL at 24m  USA (Poston et al., 2006) WL at 12m  USA (Wadden et al. 2005) WL at 4m but not at 12m  USA (Ashley et al., 2001) WL at 12m  USA (Ely et al., 2008) WL at 6m | USA (Ockene et al., 1999 – physician training only) WL at 12m  USA (Beresford et al., 1997) WL and BMI at 12m  USA (Fulkerson et al., 2015) BMI at 12m and 24m  USA (Wright et al., 2013 – boys) BMI at 12m  USA (Wilson et al., 2016) WL and BMI at 12m  USA (Cohen et al., 1991) WL at 12m  USA (Logue et al., 2005) WL unclear at 6m and non sig at 24m  USA (Franz et al., 1995) WL and BMI at 12m | USA (Christian et al.,2008) WL non sig at 12m but no of those who lost at least 6lbs sig  USA (Logsdon et al., 1989) WL at 12m but no of those who lost 5lbs or more |
| Australia / New Zealand  50% | Australia (Anderson et al., 2006) BMI at 3m  Australia (Gallagher et al., 2012) WL and BMI at 4m  New Zealand (Coppell et al., 2010) BMI but not WL at 12m | Australia (Salkeld et al., 1997 - video) WL and BMI NS at 12m  Australia (Salkeld et al., 1997 – video + self help) WL and BMI NS at 12m  Australia (Usher et al., 2013) WL and BMI at unspecified time |  |
| Asia  50% | Taiwan (Wang et al., 2012) WL at 3m | Taiwan (Huang et al., 2010) BMI at 12m |  |
| Comorbidities |  |  |  |
| Type 2 diabetes | 100% (Andrews et al., 2011) BMI at WL at 12m  100% (Coppell et al., 2010) BMI but not WL at 12m | 100% (Huang et al., 2010) BMI at 12m  100% (Franz et al., 1995) WL and BMI at 12m | 100% (Christian et al., 2008) WL non sig at 12m but no of Ps over 6lb WL sig |
| Hyperlipideamia | 100% (Ockene et al., 1999 – physician training + office support) WL at 12m | 100% (Ockene et al., 1999 – physician training only) WL at 12m |  |
| Hypertension |  | 100% (Alli et al., 1992) WL at 12m  100% (Cohen et al., 1991) WL at 12m |  |
| Various |  | Hyperlipidemia, hypertension or BMI >30 (Salkeld et al., 1997 -video) WL and BMI  Hyperlipidemia, hypertension or BMI >30 (Salkeld et al., 1997 –video + self help) WL and BMI  100% with hyperlipidemia, hypertension or type 2 diabetes (van der Veen et al., 2002) unclear if WL & BMI sig at 6m, WL & BMI ns at 12m |  |
| Cardiac conditions | 100% Cardiac conditions (Gallagher et al., 2012) WL and BMI at 4m |  |  |
| Metabolic syndrome | 100% overweight or obese (Christian et al., 2011) WL at 12m |  |  |
| Overweight / Obese  77.78% |  |  |  |
|  | 100% overweight (Martin et al., 2008) WL at 6m and 9m but not 12m and 18m  100% overweight (Ockene et al., 1999 – physician training + office support) WL at 12m  100% overweight or obese (Wadden et al., 2011 – brief lifestyle) WL at 6m but not 24m  100% overweight or obese (Wadden et al., 2011 – enhanced lifestyle) WL at 24m  100% overweight or obese (Tsai et al., 2010) WL at 6m but not at 12m  100% overweight or obese (Christian et al., 2011) WL at 12m  100% overweight or obese (Kumanyika et al., 2012) WL no with > 5% WL at 12m but not actual WL | 100% overweight (Ockene et al., 1999 – physician training only) WL at 12m | 100% overweight (Christian et al., 2008) WL non sig at 12m but no of Ps over 6lb WL sig |

Supplementary Table 3

*Effectiveness of Intervention by Intervention Characteristics for Weight Loss / Maintenance Studies*

| **Moderator** | **Significant**  **In one WL or BMI measure at any time point** | **Non significant**  **On any WL measure or BMI at any time point** | **Unclear** |
| --- | --- | --- | --- |
| Duration |  |  |  |
| Up to 9 months  65% | 1-2m (Wright et al., 2013 – girls) BMI at 12m  3m (Wang et al., 2012) WL at 3m  4m (Anderson et al., 2006) BMI at 3m  4m (Gallagher et al., 2012) WL and BMI at 4m | 1-2m (Wright et al., 2013 – boys) BMI at 12m  3m (Usher et al., 2013) WL at unspecified time |  |
|  | 6m (Wadden et al., 2011 – brief lifestyle) WL at 6m but ns at unspecified time and 24m  6m (Wadden et al., 2011 – enhanced lifestyle) WL at 24m  6m (Hacihasaoglu et al., 2011 – med compliance and education) BMI at unspecified time  6m (Leemrijse et al., 2016) BMI at 6m  6m (Ely et al., 2008) WL at 6m  6m (Tsai et al., 2010) WL sig at 6m but not at 12m  6m (Coppell et al., 2010) BMI but not WL at 12m  7m (Alkon et al., 2014) – BMI at 7m  9m (Martin et al., 2008) WL at 6m and 9m; non sig at 12m and 18m | 6m (Hacihasaoglu et al., 2011 – med adherence and questions) BMI at unspecified time  6m (Wilson et al., 2016) WL and BMI at 12m  6m (van der Veen et al., 2002) unclear if WL & BMI sig at 6m, WL & BMI ns at 12m  6m (Franz et al., 1995) WL and BMI at 12m | 6m (Andryukhin et al., 2010) BMI sig at 6m but measured as no in each cond with positive changes |
| 12 months and over  56.25% | 12m (ter Bogt et al., 2009; 2011) WL at 12m but ns at 36m  12m (Brown et al., 2011; 2014) WL at 3m but not at 6m and 12m  12m (Wadden et al., 2005) WL at 4m but not at 12m  12m (Poston et al., 2006) WL at 12m  12m (Ashley et al., 2001) WL at 12m  12m (Kumanyika et al., 2012) WL no with > 5% WL at 12m but not actual WL  12m (Andrews et al., 2011) BMI and WL at 12m  24m (Drevenhorn et al., 2012) BMI at 12m  24m (Hauptman et al, 2000) WL at 24m | 12m (Alli et al., 1992) WL at 12m  12m (Fulkerson et al., 2015) BMI at 12m and 24m  12m (Hesselink et al., 2015) WL and BMI at 24m  12m (Cohen et al., 1991) WL at 12m  12m (Huang et al., 2010) BMI at 12m  24m (Logue et al., 2005) WL unclear at 6m and non sig at 24m | 12m (Christian et al., 2008) sig at 12m for WL of over 6lbs but not for actual WL; BMi non sig |
| Number of sessions |  |  |  |
| Under 6  42.86% | 1 (Sacerdote et al., 2006) BMI at 12m but not WL  1 (Ockene et al., 1999 – physician training + office support) WL at 12m  2 (Christian et al., 2011) WL sig at 12m  4 (ter Bogt et al., 2009; 2011) WL at 12m but not 36m  4 (Andrews et al., 2011)WL and BMI at 12m  4-6 (Leemrijse et al., 2016) BMI at 6m | 1 (Ockene et al., 1999 – physician training) WL at 12m  1 (Beresford et al., 1997) WL and BMI at 12m  1 (Huang et al., 2010) BMI at 12m  2 (Logue et al., 2005) WL unclear at 6m, ns at 24m  1-3 (van der Veen et al., 2002) unclear if WL & BMI sig at 6m, WL & BMI ns at 12m  3 (Franz et al., 1995) WL and BMI at 12m | 4 (Christian et al., 2008) WL sig at 12m if no of people who lost > 6lb but not actual WL  1 (Logsdon et al., 1989) WL at 12m sig but no who lost 5lbs or more |
| 6 – 10  61.54% | 6 (Martin et al., 2008) WL at 6m and 9m; ns at 12m  6 (Hacihasaoglu et al., 2011 – med compliance and education) BMI at unspecified time  6 (Wang et al., 2012) WL at 3m  8 (Wadden et al., 2011 – brief lifestyle) WL at 6m; not at 24m  8 (Wadden et al., 2011 – enhanced lifestyle) WL at 24m  8 (Wadden et al., 2005) WL at 4m but not at 12m  8 (Tsai et al., 2010) WL at 6m but not at 12m  8 (Coppell et al., 2010) BMI but not WL at 12m | 6 (Alli et al., 1991) WL at 12m  6 (Hacihasaoglu et al., 2011 – med adherence and questions) BMI at unspecified time  6 (Vermunt et al., 2011; 2012) WL and BMI at 30m  6 (Wilson et al., 2016) WL and BMI at 12m  10 (Fulkerson et al., 2015) BMI at 12m |  |
| 10 plus  60% | 12 (Wright et al. 2013 – girls) BMI at 12m  12 (Poston et al., 2006) WL at 12m  12 (Kumanyika et al., 2012) WL no with > 5% WL at 12m but not actual WL  14 (Anderson et al., 2006) BMI at 3m  18 (Brown et al., 2011; 2014) WL at 3m but not at 6 and 12m  26 (Ashley et al., 2001) WL at 12m | 10+ (Kokkvoll et al., 2015) BMI at 24m  12 (Wright et al. 2013 – boys) BMI at 12m  12 (Cohen et al., 1991) WL at 12m | 28 (Andryukhin et al., 2010) BMI sig at 6m but measured as no in each cond with positive changes |
| Tailored |  |  |  |
| Yes | (Martin et al., 2008) WL at 6m and 9m; ns at 12m  (Sacerdote et al., 2006) BMI at 12m but not WL  (Drevenhorn et al., 2012) BMI at 24m  (Christian et al., 2012) WL at 12m | (Valve et al., 2013) BMI at unspecified time  (van der Veen et al., 2002) WL and BMI at 6m but unclear if sig; ns at 12m  (Huang et al., 2010) BMI at 12m | (Christian et al., 2008) WL sig at 12m if no of people who lost > 6lb but not actual WL  (Andryukhin et al., 2010) BMI sig at 6m but measured as no in each cond with positive changes |
| No |  | (Salkeld et al., 1997 – video) WL and BMI at 12m  (Salkeld et al., 1997 – video + self help) WL and BMI at 12m |  |
| Setting |  |  |  |
| Clinical  51.72% | GP clinic (Martin et al., 2008) WL at 6m but not at 12m or 18m  GP clinic (Sacerdote et al., 2006) BMI at 12m but not WL  GP clinic (Ockene et al., 1999 – physician training and office support) WL at 12m  GP clinic (ter Bogt et al., 2009; 2011) WL at 12m but not at 36m  Clinic (Wadden et al., 2001 – brief life style) WL at 12m but not at 24m  Clinic (Wadden et al., 2001 – enhanced life style) WL at 24m  Clinic (Kumanyika et al., 2012) WL no with > 5% WL at 12m but not actual WL  Clinic (Tsai et al., 2010) WL at 6m but not 12m  Clinic (Christian et al., 2011) WL at 12m  Primary care (Appel et al., 2011 – in person support) WL at 24m  Primary care (Appel et al., 2011 – remote support) WL at 24m  Primary care (Hauptman et al., 2000) WL at 24m  Health centre (Wang et al., 2012) WL at 3m  Community mental health (Brown et al., 2011; 2014) WL at 3m but not at 6m and 12m  Primary care / home (Hacihasaoglu et al., 2011 – med compliance and education) BMI at unspecified time | GP clinic (van der Veen et al., 2002) unclear if WL & BMI sig at 6m, WL & BMI ns at 12m  GP clinic (Ockene e al., 1999 – physician training only) WL at 12m  GP clinic (Beresford et al., 1997) WL and BMI at 12m  GP clinic (Salkeld et al., 1997 – video) WL and BMI at 12m  GP clinic (Salkeld et al., 1997 – video + self help) WL and BMI at 12m  GP clinic (Alli et al., 1992) WL at 12m  Community / inpatient (Kokkvoll et al., 2015) BMI at 24m  Vaccination centre (Valve et al., 2013) BMI at unspecified time  Primary care / home (Hacihasaoglu et al., 2011 – med adherence and questions) BMI at unspecified time  Primary care (Hesselink et al., 2015) WL and BMI at 12m  Primary care (Vermunt et al., 2001; 2012) WL and BMI at 30m | GP clinic (Christian et al., 2008) WL sig at 12m if no of people who lost > 6lb but not actual WL  Primary care (Andryukhin et al., 2010) BMI sig at 6m but measured as no in each cond with positive changes  GP clinic (Logsdon et al., 1989) WL sig at 12m but no who lost 5lbs or more |
| Non clinical  55.56% | Childcare centre (Alkon et al., 2014) BMI at 12m  School (Wright et al., 2013 – girls) BMI at 12m  School (Lemon et al., 2014) BMI at 24m  Telephone (Leemrijse et al., 2015) BMI at 6m  Research centre (Poston et al., 2006) WL at 12m | Home (Fulkerson et al., 2015) BMI at 12m  Home (Goodman et al., 2008) BMI at unspecified time  School (Wright et al., 2013 – boys) BMI at 12m  Workplace (Wilson et al., 2016) WL and BMI at 12m |  |
| Type of HCP |  |  |  |
| GP/ physician  47.06% | GP (Martin et al., 2008) WL at 6m and 9m, but not at 12m and 18m  GP (Sacerdote et al., 2006) BMI at 12m but not WL  GP (Ockene et al., 1999 – physician training + office support) WL at 12m  Physician (Appel et al., 2011 – in person support) – WL at 24m  Physician (Appel et al., 2011 – remote support) – WL at 24m  Physician (Hauptman et al., 2000) WL at 24m  Physician (Wadden et al., 2005) WL at 4m but not at 12m  Physician (Ely et al., 2008) WL at 6m | GP (Ockene et al., 1999 – physician training) WL at 12m  GP (Beresford et al., 1997) WL and BMI at 12m  GP (Salkeld et al., 1997 – video) WL and BMI at 12m  GP (Salkeld et al., 1997 – video + self help) WL and BMI at 12m  GP (Alli et al., 1992) WL at 12m  Physician (Cohen et al 1991) WL at 12m  GP (van der Veen et al., 2002) unclear if WL & BMI sig at 6m, WL & BMI ns at 12m | GP (Christian et al., 2008) - non sig actual weight loss / BMI at 12m but more had over 6lbs weight loss in intervention than comparison group  GP (Logsdon et al., 1989) WL at 12m sig but no who had lost over 6lbs or more |
| Nurse  60% | Nurse (Alkon et al., 2014) BMI at 7m  Nurse (Wright et al., 2013 – girls) BMI at 12m  Nurse (Anderson et al., 2006) BMI at 3m  Nurse (Drevenhorn et al., 2012) BMI at 24m  Nurse (Hacihasaoglu et al., 2011 – medication compliance + education) BMI at unspecified time  Nurse (Leemrijse et al., 2016) BMI at 6m  Nurse (Lemon et al., 2014) WL and BMI at 24m  Nurse practitioner (ter Bogt et al, 2009; 2011) WL at 12m but not at 36m  Public health nurse (Wang et al., 2012) WL at 3m | Nurse (Valve et al., 2013) BMI at unspecified time  Nurse (Wright et al., 2013 – boys) BMI at 12m  Nurse (Goodman et al., 2008) BMI at unspecified time  Nurse (Hacihasaoglu et al., 2011 – medication adherence + questions) BMI at unspecified time  Nurse (Hesselink et al., 2015) WL and BMI at 24m  Mental health nurse (Usher et al., 2013) WL and BMI at unspecified time |  |
| Dietitian  50% | Dietitian (Ashley et al., 2001) WL at 12m  Dietitian (Andrews et al., 2011) WL and BMI at 12m  Dietitian (Coppell et al., 2010) BMI but not WL at 12m | Dietitian (Logue et al., 2005) WL at 6m unclear if sig; ns at 24m  Dietitian (Huang et al., 2010) BMI at 12m  Dietitian (Franz et al., 1995) BMI and WL at 12m |  |
| Unspecified  100% | Primary care provider (Wadden et al., 2011 – brief lifestyle) WL at 6m but not 24m  Primary care provider (Wadden et al., 2011 – enhanced lifestyle) WL at 6m but not 24m  Primary care provider (Kumanyika et al., 2012) WL no with > 5% WL at 12m but not actual WL  Primary care provider (Tsai et al., 2010) WL at 6m but not at 12m  Primary care provider (Christian et al., 2011) WL at 12m |  |  |
| Various  37.5% | Nurse, occupational therapist, dietitian (Brown et al., 2011; 2014) WL at 3m  Exercise physiologist, & nurse (Gallagher et al., 2012) WL and BMI at 4m  Primary care physician, nurse & dietitian (Poston et al., 2006) WL at 12m | Nurse & dietitian (Fulkerson et al., 2015) BMI at 12m  Nurse & dietitian (Wilson et al., 2016) WL at BMI at 12m  Nurse & physiotherapist (Kokkvoll et al., 2015) BMI at 24m  GP, nurse practitioner, dietitian & physiotherapist (Vermunt et al., 2001; 2012) WL and BMI at 30m | Nurse & physiotherapist (Andryukhin et al., 2010) BMI sig at 6m but measured as no in each cond with positive changes |
| Comparison group |  |  |  |
| Alternative intervention  52.17% | Sham intervention (Sacerdote et al., 2006) BMI at 12m but not WL  Self directed WL programme (Appel et al., 2011 – in person support) – WL at 24m  Self directed WL programme (Appel et al., 2011 – remote support) – WL at 24m  Info on hospital / community programmes (Gallagher et al., 2012) WL and BMI at 4m  Educational leaflets (Lemon et al., 2014) WL and BMI at 24m  Intervention programme (Wang et al., 2012) WL at 3m  Weight loss counselling visits (Poston et al., 2006) WL at 12m  Sibutramine (Wadden et al., 2005) WL at 4m but not at 12m  Counselling or visits with nurse / BP + meal replacement (Ashley et al., 2001) WL at 12m  PCP training only (Ely et al., 2008) WL at 6m  Basic lifestyle intervention (Kumanyika et al., 2012) WL no with > 5% WL at 12m but not actual WL  Dietary advice (Coppell et al., 2010) BMI but not WL at 12m | Feedback on CVD risk factors + usual care (Salkeld et al., 1997 – video) WL and BMI at 12m  Feedback on CVD risk factors + usual care (Salkeld et al., 1997 – video + self help) WL and BMI at 12m  Newsletter (Fulkerson et al., 2015) BMI at 12m  Counselling (Kokkvoll et al., 2015) BMI at 24m  Sexual health counselling (Valve et al., 2013) BMI at unspecified time  Healthy lifestyle booklet (Usher et al., 2013) WL and BMI at unspecified time  Info on type 2 diabetes risk & healthy lifestyle (Vermunt et al., 2001; 2012) WL and BMI at 30m  Visit with dietitian (Franz et al., 1995) BMI and WL at 12m  UC and dietary principles summary (Huang et al., 2010) BMI at 12m | Health education materials (Christian et al., 2008) - non sig actual weight loss / BMI at 12m but more had over 6lbs weight loss in intervention than comparison group  Basic education (Andryukhin et al., 2010) BMI sig at 6m but measured as no in each cond with positive changes |
| Usual care  57.14% | UC (Martin et al., 2008) WL at 6m and 9m, but not at 12m and 18m  UC (Ockene et al., 1999 – physician training + office support) WL at 12m  UC (Wadden et al., 2011 – brief lifestyle) WL at 6m but not 24m  UC (Wadden et al., 2011 – enhanced lifestyle) WL at 6m but not 24m  UC (Brown et al., 2011; 2014) WL at 3m  UC (Drevenhorn et al., 2012) BMI at 24m  UC (Leemrijse et al., 2016) BMI at 6m  UC (ter Bogt et al, 2009; 2011) WL at 12m but not at 36m  UC (Tsai et al., 2010) WL at 6m but not at 12m  UC (Christian et al., 2011) WL at 12m  Usual school PA programme (Wright et al., 2013 – girls) BMI at 12m  UC (Andrews et al., 2011) WL and BMI at 12m | UC (Logue et al., 2005) WL at 6m unclear if sig; ns at 24m  UC (van der Veen et al., 2002) unclear if WL & BMI sig at 6m, WL & BMI ns at 12m  UC (Ockene et al., 1999 – physician training) WL at 12m  UC (Beresford et al., 1997) WL and BMI at 12m  UC (Alli et al., 1992) WL at 12m  UC (Goodman et al., 2008) BMI at unspecified time  UC (Hesselink et al., 2015) WL and BMI at 24m  UC (Cohen et al 1991) WL at 12m  Usual school PA programme (Wright et al., 2013 – boys) BMI at 12m | UC (Logsdon et al., 1989) WL at 12m sig but no who had lost 5lbs or more |
| Delayed intervention / measurement only  60% | Delayed intervention (Alkon et al., 2014) BMI at 7m  Measurement only (Anderson et al., 2006) BMI at 3m  Measurement only (Hacihasaoglu et al., 2011 – medication compliance + education) BMI at unspecified time | Measurement only (Hacihasaoglu et al., 2011 – medication adherence + questions) BMI at unspecified time  Measurement only (Wilson et al., 2016) WL at BMI at 12m |  |
| Placebo | (Hauptman et al., 2000) WL at 24m |  |  |
| Theory |  |  |  |
| Theory used |  | TTM (van der Veen et al., 2002) unclear if WL & BMI sig at 6m, WL & BMI ns at 12m  SLT (Beresford et al., 1997) WL & BM at 12m |  |
| Theory not used |  |  |  |
| BCTs used |  |  |  |
| Goal setting  62.5% | (Anderson et al., 2006) BMI at 3m  (Brown et al., 2011; 2014) WL at 3m  (Leemrijse et al., 2016) BMI at 6m  (ter Bogt et al, 2009; 2011) WL at 12m but not at 36m  (Kumanyika et al., 2012) WL no with > 5% WL at 12m but not actual WL | (Fulkerson et al., 2015) BMI at 12m  (Wilson et al., 2016) WL at BMI at 12m | (Christian et al., 2008) - non sig actual weight loss / BMI at 12m but more had over 6lbs weight loss in intervention than comparison group |
| Goal review  0% |  |  | (Christian et al., 2008) - non sig actual weight loss / BMI at 12m but more had over 6lbs weight loss in intervention than comparison group |
| Pharmacological support  100% | (Wadden et al., 2011 – enhanced lifestyle) WL at 6m but not 24m  (Drevenhorn et al., 2012) BMI at 24m  (Hauptman et al., 2000) WL at 24m  (Poston et al., 2006) WL at 12m  (Wadden et al., 2005) WL at 4m but not at 12m |  |  |
| Action planning  100% | (Anderson et al., 2006) BMI at 3m  (Leemrijse et al., 2016) BMI at 6m |  |  |
| Self monitoring  77.78% | (Anderson et al., 2006) BMI at 3m  (Leemrijse et al., 2016) BMI at 6m  (Wang et al., 2012) WL at 3m  (Wadden et al., 2005) WL at 4m but not at 12m  (Kumanyika et al., 2012) WL no with > 5% WL at 12m but not actual WL  (Tsai et al., 2010) WL at 6m but not at 12m  (Andrews et al., 2011) WL and BMI at 12m | (Vermunt et al., 2001; 2012) WL and BMI at 30m  (Wilson et al., 2016) WL at BMI at 12m |  |
| Instructions  0% |  |  | (Andryukhin et al., 2010) BMI sig at 6m but measured as no in each cond with positive changes |
| Feedback  100% | (Brown et al., 2011; 2014) WL at 3m  (Leemrijse et al., 2016) BMI at 6m  (ter Bogt et al, 2009; 2011) WL at 12m but not at 36m |  |  |

Supplementary Table 4

*Effectiveness of Intervention by Quality for Weight Loss / Maintenance Studies*

| **Moderator** | **Significant WL (on at least one measure), BMI or dietary behaviour (on at least one measure)** | **Non-significant** | **Unclear** |
| --- | --- | --- | --- |
| Risk of Bias |  |  |  |
| Low  58.82% | Obesity guidelines (Wadden et al., 2011 – brief lifestyle) WL at 6m but not at 24m  Obesity guidelines (Wadden et al., 2011 – enhanced lifestyle) WL at 24m  Obesity guidelines (Kumanyika et al., 2012) number with 5% BODY WEIGHT at 12m but WL non sig  Obesity guidelines (Tsai et al., 2010) WL at 6m but not 12m  Obesity guidelines (Christian et al., 2011) WL at 12m  Jadad 4/4 (Hacihasaoglu et al., 2011 – med enhancement + education) BMI at unspecified time  Jadad 4/4 (ter Bogt et al., 2009; 2011) WL at 12m but not 36m  CONSORT good (Wadden et al., 2005) WL at 4m but not at 12m  Cochrane low (Andrews et al., 2011) BMI and WL at 12m  Cochrane low (Coppell et al., 2010) BMI but not WL at 12m | QCC Overall (Beresford et al., 1997) WL & BMI at 12m  Jadad 4/4 (Fulkerson et al., 2015) BMI at 12m  Jadad 4/4 (Goodman et al., 2008) BMI at unspecified time  Jadad 4/4 (Hacihasaoglu et al., 2011 – med adherence + questions) BMI at unspecified time  Jadad 4/4 (Hesselink et al., 2015) WL & BMI at 24m  Cochrane low (Huang et al., 2010) BMI at 12m | QCC Overall / CONSORT good (Christian et al., 2008) WL sig but measure was lost 6lbs or more; BMI, and WC non sig |
| Unclear / fair / moderate  47.83% | QCC Overall / CONSORT fair (Martin et al., 2008) WL sig at 6 & 9m but not at 12 & 18m  QCC Overall (Sacerdote et al., 2006) BMI sig at 12m but not WL  QCC Overall / fair CONSORT (Ockene et al., 1999 – physician training + office support) WL at 12m  Jadad 3/4 (Gallagher et al., 2012) WL & BMI at 4m  Jadad 3/4 (Leemrijse et al., 2016) BMI at 6m  Jadad 3/4 (Lemon et al., 2014) WL & BMI at 24m  Jadad 3/4 (Wang et al., 2012) WL at 3m  CONSORT fair (Hauptman et al., 2000) WL at 24m  CONSORT fair (Poston et al., 2006) WL at 12m  CONSORT fair (Ashley et al., 2001) WL at 12m  CONSORT fair (Ely et al., 2008) WL at 6m | QCC Overall (van der Veen et al., 2002) unclear if WL & BMI sig at 6m, WL & BMI ns at 12m  QCC Overall / fair CONSORT (Ockene et al., 1999 – physician training) WL at 12m  QCC Overall (Salkeld et al., 1997 – video) WL & BMI at 12m  QCC Overall (Salkeld et al., 1997 – video + self help) WL & BMI at 12m  QCC Overall (Alli et al., 1992) WL at 12m  Jadad 3/4 (Kokkvoll et al., 2015) BMI at 24m  Jadad 3/4 (Vermunt et al., 2011; 2012) WL & BMi at 30m  CONSORT fair (Cohen et al., 1991) WL at 12m  CONSORT fair (Logue et al., 2005) WL at 6m unclear if sig; ns at 24m  Cochrane unclear (Franz et al., 995) BMI and WL at 12m | Jadad 3/4 (Andryukhin et al., 2010) BMI sig but measured as no in each cond with positive changes  QCC Overall (Logsdon et al., 1989) WL at 12m sig but no who had lost 5lbs or more |
| High  55.56% | Jadad 2/4 (Alkon et al., 2014) BMI at 7m  Jadad 2/4 (Wright et al., 2013 – girls) BMI at 12m  Jadad 2/4 (Anderson et al., 2006) BMI at 3m  Jadad 2/4 (Brown et al., 2011; 2014) WL at 3m but not at 6m  Jadad 2/4 (Drevenhorn et al., 2012) BMI at 24m | Jadad 2/4 (Valve et al., 2013) BMI at unspecified time  Jadad 2/4 (Wright et al., 2013 – boys) BMI at 12m  Jadad 1/4 (Usher et al., 2013) WL & BMI at unspecified time  Jadad 1/4 (Wilson et al., 2016) WL & BMI at 12m |  |
| Not reported  100% | NR (Appel et al., 2011 – in person support) WL at 12m  NR (Appel et al., 2011 – remote support) WL at 12m |  |  |

The citation for the Synthesis Without Meta-analysis explanation and elaboration article is: Campbell M, McKenzie JE, Sowden A, Katikireddi SV, Brennan SE, Ellis S, Hartmann-Boyce J, Ryan R, Shepperd S, Thomas J, Welch V, Thomson H. Synthesis without meta-analysis (SWiM) in systematic reviews: reporting guideline BMJ 2020;368:l6890 <http://dx.doi.org/10.1136/bmj.l6890>

| **SWiM is intended to complement and be used as an extension to PRISMA** | | | |
| --- | --- | --- | --- |
| **SWiM reporting item** | **Item description** | **Page in manuscript where item is reported** | **Other*** |
| *Methods* | | | |
| **1** Grouping studies for synthesis | 1a) Provide a description of, and rationale for, the groups used in the synthesis (e.g., groupings of populations, interventions, outcomes, study design) | 7-8 |  |
|  | 1b) Detail and provide rationale for any changes made subsequent to the protocol in the groups used in the synthesis | 6 |  |
| **2** Describe the standardised metric and transformation methods used | Describe the standardised metric for each outcome. Explain why the metric(s) was chosen, and describe any methods used to transform the intervention effects, as reported in the study, to the standardised metric, citing any methodological guidance consulted | 7 (definition of a successful intervention) |  |
| **3** Describe the synthesis methods | Describe and justify the methods used to synthesise the effects for each outcome when it was not possible to undertake a meta-analysis of effect estimates | 7-8 |  |
| **4** Criteria used to prioritise results for summary and synthesis | Where applicable, provide the criteria used, with supporting justification, to select the particular studies, or a particular study, for the main synthesis or to draw conclusions from the synthesis (e.g., based on study design, risk of bias assessments, directness in relation to the review question) | 6-7 |  |
| **SWiM reporting item** | **Item description** | **Page in manuscript where item is reported** | **Other*** |
| **5** Investigation of heterogeneity in reported effects | State the method(s) used to examine heterogeneity in reported effects when it was not possible to undertake a meta-analysis of effect estimates and its extensions to investigate heterogeneity | 7-8 |  |
| **6** Certainty of evidence | Describe the methods used to assess certainty of the synthesis findings | 8-7 |  |
| **7** Data presentation methods | Describe the graphical and tabular methods used to present the effects (e.g., tables, forest plots, harvest plots).  Specify key study characteristics (e.g., study design, risk of bias) used to order the studies, in the text and any tables or graphs, clearly referencing the studies included | Table 1-4; p. 9-10 |  |
| *Results* | | | |
| **8** Reporting results | For each comparison and outcome, provide a description of the synthesised findings, and the certainty of the findings. Describe the result in language that is consistent with the question the synthesis addresses, and indicate which studies contribute to the synthesis | p. 10-12 |  |
| *Discussion* |  |  |  |
| **9** Limitations of the synthesis | Report the limitations of the synthesis methods used and/or the groupings used in the synthesis, and how these affect the conclusions that can be drawn in relation to the original review question | 14 |  |

PRISMA=Preferred Reporting Items for Systematic Reviews and Meta-Analyses.

*If the information is not provided in the systematic review, give details of where this information is available (e.g., protocol, other published papers (provide citation details), or website (provide the URL)).
